# An upper left lateral incisor with double roots and double root canals: A case report

**DOI:** 10.1097/MD.0000000000042815

**Published:** 2025-06-27

**Authors:** Zhou-Bin Xia, Jing Ren, Jia-Xiang Chen, Yu Wei, Yan Yan, Liang-Ju Cao

**Affiliations:** a Department of Stomatology, The First People’s Hospital of Kunming, Kunming, Yunnan Province, China; b Kunming Medical University Haiyuan College, Kunming, Yunnan Province, China.

**Keywords:** double root, double root canal, maxillary lateral incisors, root canal treatment, root canal variations

## Abstract

**Rationale:**

Most maxillary lateral incisors have only 1 root and 1 root canal; the presence of both double roots and double canals is extremely rare and can lead to persistent symptoms if not properly diagnosed.

**Patient concerns:**

A 36-year-old female presented with a 3-month history of discomfort in the upper left anterior tooth, following prior root canal treatment performed 2 years earlier for pulp necrosis.

**Diagnoses:**

Cone beam computed tomography imaging revealed a left maxillary lateral incisor (tooth 22) with double roots and double root canals, with chronic apical periodontitis associated with the untreated palatal canal.

**Interventions:**

The patient underwent root canal retreatment involving the removal of old filling material, negotiation and preparation of the missed palatal canal using manual and rotary files, calcium hydroxide dressing, and final obturation with cold lateral compaction and gutta-percha.

**Outcomes:**

The patient’s symptoms resolved completely after retreatment, and no discomfort was reported during a 1-year follow-up.

**Lessons:**

Dental practitioners should possess a comprehensive understanding of the typical anatomy of the dental root canal system and remain cognizant of the potential variations in root canal configurations. When root canal variation is suspected, meticulous examination of the pulp chamber floor, altering the X-ray projection angle, or conducting a high-resolution cone beam computed tomography scan is essential to ensure accurate diagnosis and prevent missed canals, ultimately improving treatment success.

## 1. Introduction

Apical periodontitis is an inflammatory response in the periapical tissues, typically triggered by bacterial biofilms within the root canal system. Adequate cleaning, shaping, disinfection, and sealing of the root canal system are decisive factors in successful root canal treatment of teeth with apical periodontitis. These steps are important to control infection, prevent further re-infection of the root canal system, and establish environmental conditions conducive to periapical tissue healing.^[1]^ The maxillary lateral incisor exhibits significant morphological variability, often appearing reduced in size but occasionally as large as a central incisor. It can have diverse crown shapes, including peg-shaped, cone-shaped, barrel-shaped, and canine-shaped forms. Additionally, interruption grooves and deep lingual pits are more common in lateral incisors than in central incisors. These variations result from genetic, epigenetic, and environmental influences. The maxillary lateral incisor often has a single root canal, but the occurrence of a double root canal is less common.^[2]^ The prevalence of double root canals in maxillary lateral incisors is around 3%, with an even lower incidence of double roots and double canals.^[1]^ In a study, it was revealed that when root canal treatment and apical sealing are inadequate, the risk of post-treatment apical periodontitis is significantly increased.^[3]^ Anatomical variations often lead to missed canals during endodontic treatment, which can compromise the outcome of apical periodontitis management. Therefore, a thorough understanding of the complex anatomy of the root canal system is crucial for effective treatment.

Cone beam computed tomography (CBCT), which provides low-radiation imaging, has become a commonly used diagnostic tool for dental diseases in recent years.^[4]^ Conventional radiography is a practical and cost-effective method for obtaining 2-dimensional images of root canal structures but may suffer from distortions and limited detail. Periapical radiographs can only provide 2-dimensional information and may not be able to detect additional root canals, especially a variation of a root with buccolingual direction. In contrast, CBCT offers high-resolution, 3-dimensional imaging, allowing for more accurate evaluation, diagnosis, treatment planning, and post-treatment follow-up while being less error-prone than periapical radiographs.^[5,6]^ Among various radiographic techniques compared for accuracy, CBCT has demonstrated the highest precision in identifying root canal morphology. As such, it serves as a valuable diagnostic tool in endodontics, aiding in treatment planning and outcome assessment, especially in cases where conventional 2D imaging may not reveal the full complexity of the canal system. In addition, CBCT has been used in several studies to establish a clear association between untreated root canals and post-treatment apical periodontitis.^[7,8]^

In this report, we present a case concerning a patient who underwent root canal treatment for her maxillary lateral incisor at a different medical facility and continued to experience prolonged discomfort thereafter. Untreated root canal was not detected in the palatal side in the periapical X-rays provided by the hospital and the initial periapical X-ray conducted in our hospital. Utilizing the CBCT, the patient was diagnosed with the left maxillary lateral incisor with double roots and a double root canal system. After thorough root canal retreatment, including locating the missed root canal, enlarging with files, irrigating for disinfection, and completing the root canal filling, the painful symptoms of the patient subsided.

## 2. Case statement

**Figure 1.**
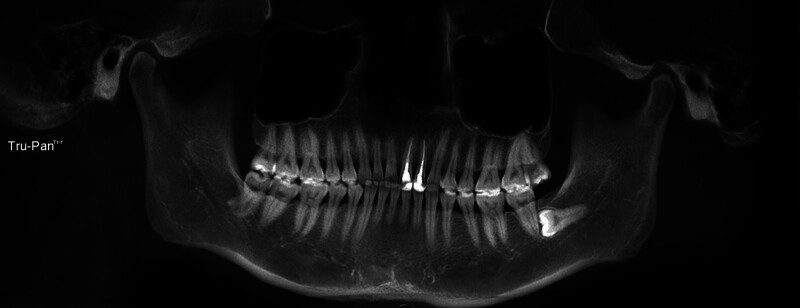
CBCT panorama. CBCT = cone beam computed tomography.

**Figure 2.**
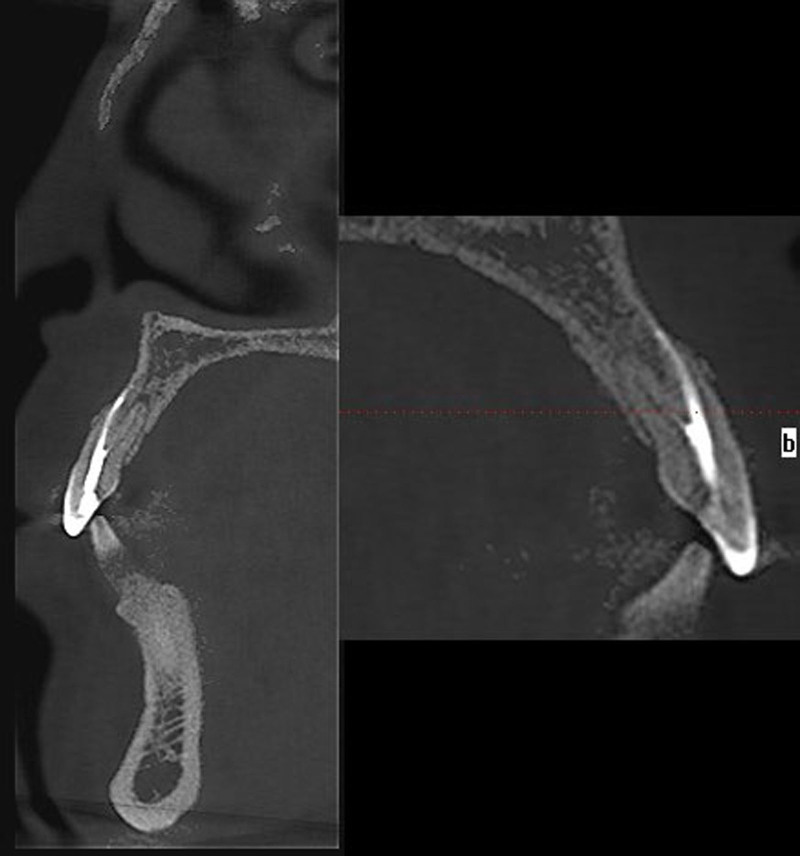
Coronal/sagittal view.

**Figure 3.**
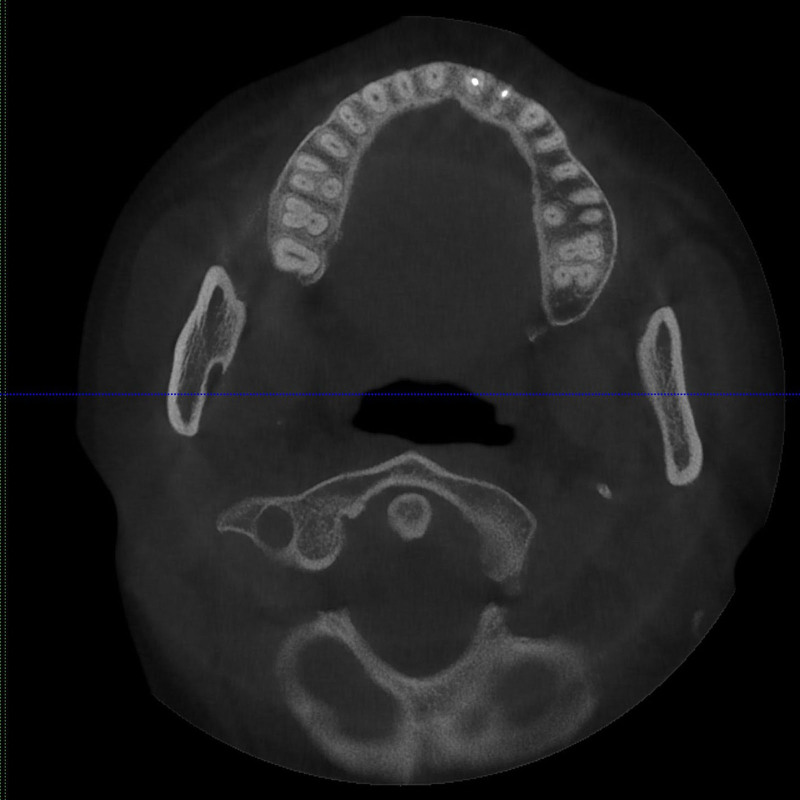
Maxillary dentition in horizontal view.

**Figure 4.**
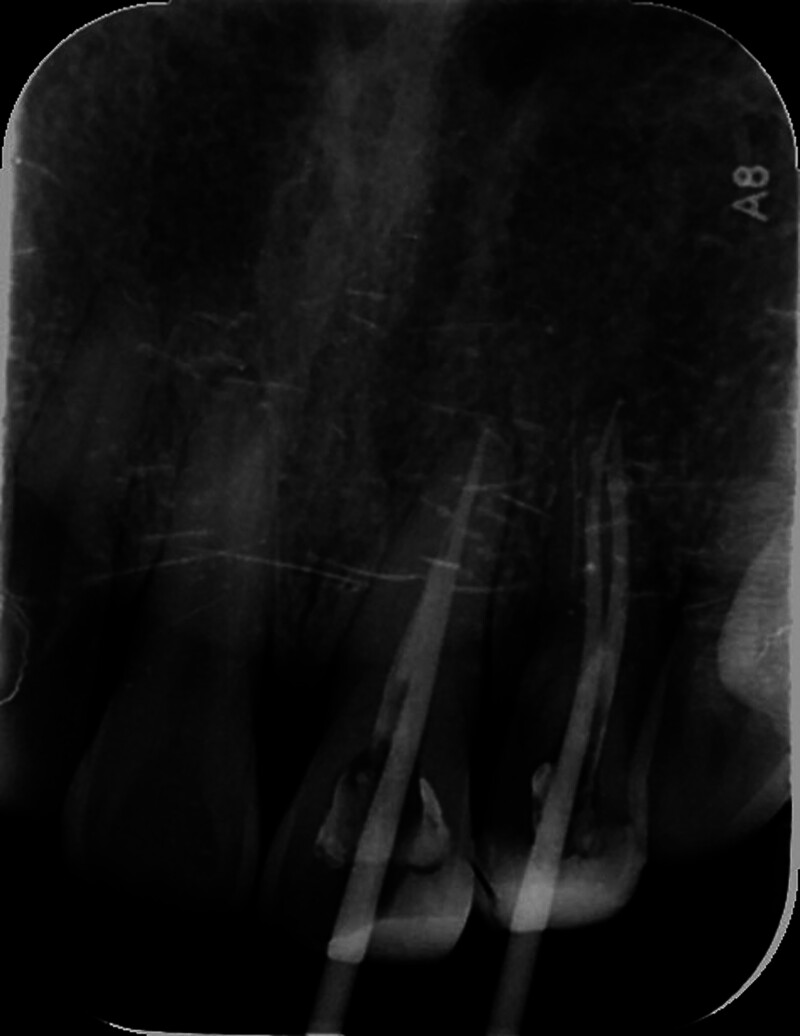
Imaging used to assist in calibrating the primary root tip.

**Figure 5.**
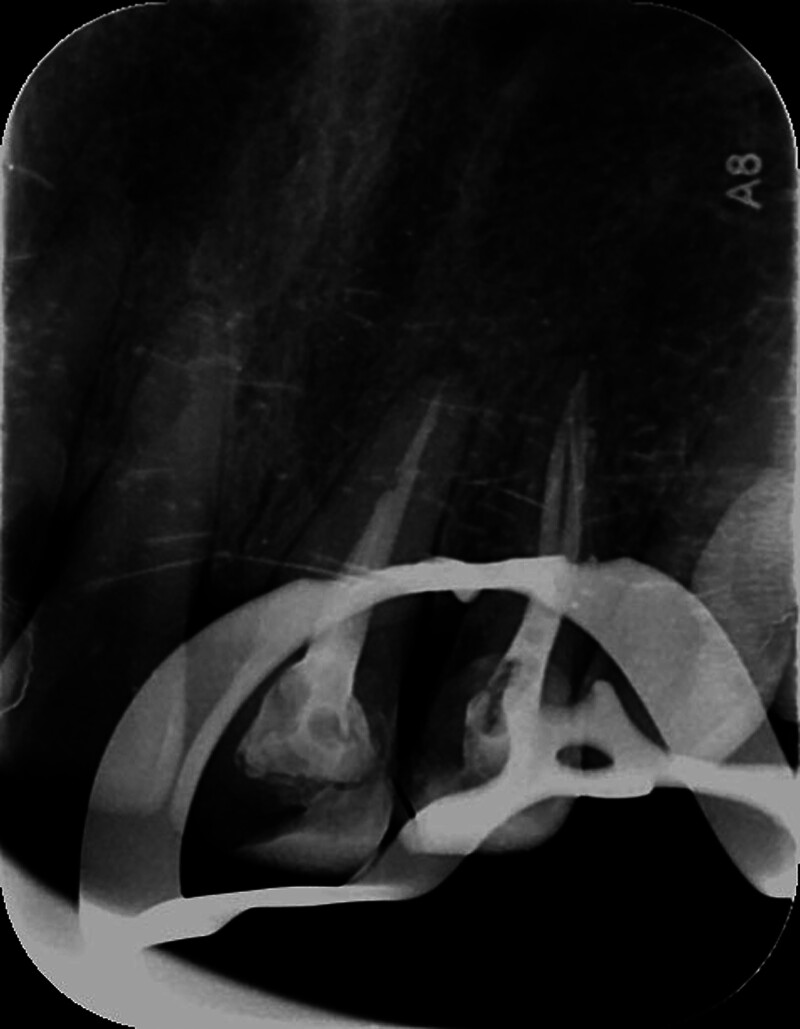
Imaging after root canal was filled.

A female patient, aged 36, presented to the hospital with a primary concern of experiencing discomfort in the upper left anterior tooth persisting for a duration of 3 months. Two years ago, the patient underwent standard root canal treatment at an external clinic due to dental caries leading to pulp necrosis. Since then, she had consistently experienced discomfort during biting and occasional spontaneous pain. X-ray examination did not reveal any missing or untreated root canals in the teeth. However, no CBCT examination or follow-up was conducted. The patient had undergone root canal treatment for her upper left front tooth following a traumatic incident 5 months prior. Intraoral examination revealed that the incisal 1/3 of the tooth with the dental notation of 22 had a tooth-colored filling, no pain on percussion, no mobility, and no abnormality detected on periodontal examination. A panoramic radio-opaque image of root canals of the tooth by the CBCT scan (Kavo 3D eXam, Biberach an der Riss, Germany; power: 1 kVA, voltage: 220V ± 10%, power supply frequency: 50/60Hz) revealed that there were no obvious abnormalities around the root apex (Fig. 1), there were 2 roots on the labial and palatal sides (Fig. 2), and the image of the double root canals on the labial and palatal sides could be seen in the horizontal view (Fig. 3). High-density radio-opaque images were evident in the labial root canal, indicating a dense and compact filling. However, in the palatal root canal, no radio-opaque images were discernible (refer to Figs. 2 and 3). Furthermore, a low-density radiographic image was observed in the palatal apical region. The final diagnosis was chronic apical periodontitis with respect to the tooth with a dental notation of 22. In the first visit, preliminary negotiation of the missed canal was performed, and the root canal preparation was carried out with a manual S1 nickel-titanium file (Dentsply, Charlotte). The patient was administered the following treatment: The procedure involved the extraction of the existing filling in the tooth and the extirpation of the gutta-percha filling within the pulp chamber and canals. Subsequently, a meticulous exploration of the pulp chamber was undertaken to locate the opening of the palatal canal orifice. Utilizing a K-file (Dentsply), the palatal root canal was prepared, followed by the insertion of a calcium hydroxide (Fuji Corporation, Japan) paste into the root canal. Temporary sealing (Fuji Corporation) of both the root canal and pulp chamber was accomplished using glass ionomer restorative cement (Fuji Corporation). Subsequently, during the follow-up examination 1 week later, the palatal root canal of the tooth denoted as 22 underwent cleansing and clearance procedures to ensure unobstructed patency. The working length of the root canal was measured to be 20 mm using an endodontic scale, and the root canal was biomechanically prepared using the WaveOne endodontic rotary instruments, adhering to the step-down method. The rotary nickel-titanium root canal preparation file used in this case is a single-file system (Dentsply) with reciprocating rotation. It is designed to complete the entire preparation with a single file. In this case, a size 25 single file was used. The canal was then sealed using calcium hydroxide paste. Calcium hydroxide medication was removed by irrigation and flushing with a syringe filled with physiological saline. Cold lateral compaction was used for irrigation. One week later, the patient had no symptoms of discomfort at the follow-up visit. The apex of the root was calibrated (Fig. 4), and the root canal was filled with 6% taper gutta-percha cone (Dentsply) (Fig. 5). The pulp chamber was sealed using fluid resin as the base, and final restoration was accomplished using Z350 resin (3M Company, Saint Paul). The crown restoration procedure for the tooth was conducted on a specific date. No operation microscope or magnification was used during the treatment. One year later, the patient reported no symptoms or discomfort in a follow-up phone call. Informed consent was obtained from this patient.

This study was conducted with approval from the Ethics Committee of The First People’s Hospital of Kunming, and written informed consent was obtained from the participant.

## 3. Discussion

Most maxillary lateral incisors have only a single root and 1 root canal. The incidence of double root canals is approximately 3%, and the occurrence of a maxillary lateral incisor featuring both double roots and double root canals is even more uncommon.^[9]^ The double-root lateral incisors located distal to the facial midline can be observed through X-ray examination. However, identifying double-rooted lateral incisors in the labial and palatal orientations is challenging due to the overlapping nature of the images. The CBCT scan revealed that tooth number 22 had a bifurcated root and a bifurcated canal both in the labial and palatal directions. In this patient, only the labial root canal was filled in another hospital, while the palatal root canal was overlooked.^[10]^ Variants of the maxillary lateral incisors are common and typically manifest as conical teeth, invaginated lingual fossa, palatogingival groove, and dental invagination.^[11]^ Anatomic variations of double root canals are less common clinically, and incidences of double root canals are even rarer. Double root canals in maxillary lateral incisors can be considered as simple anatomic variations of root canals. Some special cases such as palatogingival groove, and dental invagination (Oehler type III)^[12]^ can lead to the occurrence of a single root with double root canals. Fused teeth can result in such cases with double roots and root canals.^[13]^ From the perspective of tooth development, the occurrence of a supernumerary root in double-rooted maxillary lateral incisors may be related to abnormal development of the Hertwig epithelial root sheath. During the root development process of multi-rooted teeth, the Hertwig epithelial root sheath sinks, fuses, and then separates to form multiple roots.^[14]^ From an evolutionary perspective, the occurrence of supernumerary root in the maxillary lateral incisors can be seen as an atavistic phenomenon. The evolution of teeth is from homodont and haplodont to heterodont and multi-cusped. Heterodont and multi-cusped teeth are derived from the fusion of multiple haplodont teeth during the evolutionary process.^[15]^ The crown of the human maxillary incisor is formed by the fusion of 4 developmental lobes (mesial, distal, labial, and lingual developmental lobes). The crown morphology of multi-rooted maxillary incisors is normal, as the roots do not fuse to form a single root due to changes in genetic material or developmental abnormalities, resulting in the preservation of the original root shape of multiple single canines.^[16]^ Wang et al documented a case of a maxillary central incisor with 3 roots and 3 canals. Two of the roots were located on the labial side and the remaining 1 was located under the cingulum, whose locations coincided with those of the developmental lobes of the tooth.^[17]^ In another case, a patient had a maxillary lateral incisor with 2 roots parallel along the mesial to the distal direction and similar in size.^[18]^ In this case, the roots of the left maxillary lateral incisor of the patient were along the labial to lingual direction.

In addition to dental invagination, palatogingival groove, and fused teeth, maxillary lateral incisors with double root canals can be classified into 2 types: 1 is single root with 2 root canals and the other is double roots with 2 separate root canals. Among them, a maxillary lateral incisor with single root and 2 root canals is classified as Vertucci type II (Type II–II)^[19]^ or type V (Type I–II).^[20]^ A maxillary lateral incisor with 2 roots and 2 root canals is usually oriented along the labial to palatal direction. Maxillary lateral incisors with double roots and double root canals have 3 types of root furcation position: furcation located at 1/3 the distance of the dental crown, mid-root furcation, and furcation at the root tip of the tooth. The supernumerary root is usually located on the palatal side. For a tooth with furcation located at 1/3 the distance of the dental crown, the root canal systems of the primary and the supernumerary roots may be independent of each other.^[21]^ In this case, the root bifurcation position is located at 1/3 the distance of the dental crown, which bifurcates into 2 completely independent root canals.

In the present case, the omission of the palatal root canal during the initial treatment phase led to a failure in the root canal treatment. This underscores the importance of diligently assessing X-ray films for the potential presence of double-root anatomical variations before commencing root canal treatment for the maxillary lateral incisor. If double root canals are suspected, the utilization of CBCT scans can offer a more comprehensive understanding of the root canal anatomy of the affected tooth. For maxillary lateral incisors with 1 root and double root canals, the shape of the access opening into the pulp chamber can be slightly extended to the lingual aspect of the tooth. The root canals may be probed along the palatal side of the primary root canal to detect a supernumerary root or an accessory canal. Upon gaining access to the pulp of a double-rooted maxillary lateral incisor with a bifurcation occurring at one-third of the dental crown’s length, it is crucial to recognize that the primary and accessory root canal systems may operate independently. Consequently, a distinct access opening to the pulp chamber for each root canal system may be necessary.^[16]^ Based on the aforementioned analysis, the etiology underlying the omission of the root canal in this case can be attributed to the operator’s failure to meticulously review the preoperative imaging examination. The conservative approach adopted in the access opening of the pulp chamber, without extension to the palatal side, played a pivotal role in the oversight of the palatal root canal.

The number, shape, and course of the root canals are factors that need to be evaluated before performing root canal treatment. Adequate preoperative evaluation can mitigate the omission of root canals during treatment; and prevent lateral perforations and the occurrence of ledges, thus improving the success rate of treatment. CBCT scan allows the anatomy and orientation of the root canals to be visualized in a 3-dimensional view. In this case, it was confirmed before surgery by CBCT scan that the root of the left maxillary lateral incisor has double roots and 2 root canals that were oriented along the labial to lingual direction. In the process of establishing access to the pulp chamber, attention should be paid to the location of the pulp orifice opening, and simultaneously not cause any damage to the pulp chamber floor. The shape of the pulp chamber floor helps to identify the location and number of root canals. Furthermore, in instances where a patient experiences discomfort during or after root canal treatment, and after excluding the presence of carious lesions in neighboring teeth, it is imperative to contemplate the potentiality of overlooked root canals within the implicated tooth. Increasing magnification and illumination enhance the possibility of finding all root canals during root canal treatment.^[22]^ Due to the paired nature of teeth, when a variation is found in the root canal system of 1 tooth, it should be considered that a similar variation may exist in the root canal system of the contralateral tooth. However, in this case, there was no variation in the root canal system of the contralateral tooth.

It is worth mentioning that due to the patient’s poor compliance, possibly related to the long distance between their residence and our hospital, as they live in a different province, the patient declined multiple follow-up visits for clinical and radiographic examinations. Considering China’s vast geographic territory, this is a common challenge. The patient only reported verbally that the treated tooth felt normal and caused no discomfort, assuring us that she would return to our hospital for evaluation and treatment if any symptoms arose. Based on this feedback, we believe the treatment was effective. Nonetheless, we will continue to conduct regular follow-up calls to encourage and remind the patient to attend future checkups.

This case also has its particularity, as the maxillary lateral incisor presented with both double roots and double canals oriented labio-palatally, a rare variation that led to treatment failure due to missed anatomy; therefore, it is crucial for clinicians to enhance their awareness of such variations and utilize tools like CBCT for accurate diagnosis and effective treatment.

## 4. Conclusion

This report describes a rare case of a maxillary lateral incisor with 2 roots and 2 separate root canals, highlighting the clinical challenges posed by atypical root canal anatomy. Although maxillary lateral incisors are typically considered to have a single canal, this assumption can lead to missed canals, especially in the buccolingual direction, due to the limitations of conventional radiographs. Such variations, while uncommon, are underreported in the Chinese population. In this case, the patient’s symptoms were attributed to a previously missed palatal root following initial root canal therapy. This underscores the importance of thoroughly examining the floor of the pulp chamber and considering the use of CBCT when root canal variation is suspected. CBCT offers high diagnostic accuracy in identifying complex canal morphologies, thereby guiding appropriate retreatment. Developmental anomalies involving Hertwig epithelial root sheath may play a key role in such variations. Ultimately, early recognition and proper imaging are essential to improve treatment outcomes and avoid failures related to undetected anatomical anomalies.

## Author contributions

**Conceptualization:** Zhou-Bin Xia, Liang-Ju Cao.

**Data curation:** Zhou-Bin Xia, Jia-Xiang Chen, Yu Wei, Yan Yan.

**Funding acquisition:** Zhou-Bin Xia, Liang-Ju Cao.

**Formal analysis:** Jing Ren, Jia-Xiang Chen, Yu Wei, Yan Yan.

**Writing – review & editing:** Jing Ren, Liang-Ju Cao.

**Writing – original draft:** Yu Wei.
